# Currarino syndrome: a comprehensive genetic review of a rare congenital disorder

**DOI:** 10.1186/s13023-021-01799-0

**Published:** 2021-04-09

**Authors:** Gabriel C. Dworschak, Heiko M. Reutter, Michael Ludwig

**Affiliations:** 1 Institute of Human Genetics, Medical Faculty, University of Bonn, Venusberg-Campus 1, 53127 Bonn, Germany; 2 Institute of Anatomy and Cell Biology, Medical Faculty, University of Bonn, 53115 Bonn, Germany; 3 Department of Pediatrics, University Hospital Bonn, 53127 Bonn, Germany; 4 Department of Neonatology and Pediatric Intensive Care, University Hospital Bonn, 53127 Bonn, Germany; 5 Department of Clinical Chemistry and Clinical Pharmacology, University of Bonn, 53127 Bonn, Germany

**Keywords:** Anorectal malformation, Constipation, Currarino syndrome, *MNX1*, Presacral mass, Sacral agenesis

## Abstract

**Background:**

The triad of a presacral mass, sacral agenesis and an anorectal anomaly constitutes the rare Currarino syndrome (CS), which is caused by dorsal–ventral patterning defects during embryonic development. The major causative CS gene is *MNX1*, encoding a homeobox protein.

**Main body:**

In the majority of patients, CS occurs as an autosomal dominant trait; however, a female predominance observed, implies that CS may underlie an additional mode(s) of inheritance. Often, the diagnosis of CS is established solely by clinical findings, impacting a detailed analysis of the disease. Our combined data, evaluating more than 60 studies reporting patients with CS-associated mutations, revealed a slightly higher incidence rate in females with a female-to-male ratio of 1.39:1. Overall, *MNX1* mutation analysis was successful in only 57.4% of all CS patients investigated, with no mutation detected in 7.7% of the familial and 68% of the sporadic patients. Our studies failed to detect the presence of an expressed *MNX1* isoform that might explain at least some of these mutation-negative cases.

**Conclusion:**

Aside from *MNX1*, other genes or regulatory regions may contribute to CS and we discuss several cytogenetic studies and whole-exome sequencing data that have implicated further loci/genes in its etiology.

**Supplementary Information:**

The online version contains supplementary material available at 10.1186/s13023-021-01799-0.

## Background

The first report of the syndrome has been published by Roger L. J. Kennedy in 1926 [1]. However, the description of the triad as a unique entity was defined by Guido Currarino and colleagues in 1981 [2] and hence, the term “Currarino syndrome” (CS, OMIM #176450) was coined [3]. Yates and coworkers were the first to describe an autosomal dominant mode of inheritance [4].

Three main features are characteristics of CS: (1) anterior sacral bone defects, known as sacral scimitar or sickle-shaped sacrum, or a complete sacral agenesis below S2; (2) anorectal malformation (often clincically present as chronic constipation); (3) a presacral mass, representing an anterior meningocele, a teratoma or an enteric cyst or any combination of these [2, 5–7]. Teratomas in CS are often benign however, a malignant transformation has been observed, even in childhood [6, 8, 9]. There is evidence that the risk of malignant transformation of sacrococcygeal teratoma and of presacral teratoma in CS is different despite similar position and appearance. Dirix et al. have analyzed 205 sacrococcygeal teratoma and 16 CS patients in a retrospective study [10]. Patients with sacrococcygeal teratoma had a lower malignancy-free survival than patients with a presacral teratoma associated with CS (58% versus 100% after two years). However, reliable biomarker that predict the risk of malignancy are lacking. Some authors suggested removal of the tumor, even in asymptomatic patients [11]. In 2008, Crétolle et al. suggested, that neural tube defects should also be considered as a fourth major clinical feature of CS [12]. These defects comprise tethered cord, fatty, thickened filum and/or spinal lipoma and neuroenteric cysts [12, 13].

As a further feature in association with CS, Müllerian duct anomalies (e.g. bicornuate uterus, septate vagina, agenesis or hypoplasia of uterus and vagina) have been found in up to 15% of cases [13, 14]. Additional genitourinary findings in CS comprise horseshoe or duplex kidney, vesicoureteral reflux, neurogenic bladder, recurrent urinary tract infections, urinary incontinence and hydronephrosis [13]. Developmental delay may also be present and is a clue to a cytogenetic anomaly involving 7q36.3 [12]. As CS shows variable expressivity, patients may either be recognized with the complete spectrum or only components of the four major features. Even complete asymptomatic mutation carriers have been reported [13]. Mutations affecting the *MNX1* gene on chromosome 7q36.3 [14], encoding the motor neuron and pancreas homeobox protein 1, have been detected in most of the familiar cases and in about one third of sporadic CS patients [13, 16, 17].

In this review, we focus on the current knowledge of CS etiology and the genetic findings in the context of this rare disease.

## Main text

### Penetrance and prevalence of CS

The clinical phenotype of CS is extremely variable and the classic triad often incompletely present, even within the same family. Even patients with causative heterozygous *MNX1* mutations, may present with only one or two classical features and moreover, chronic constipation since early childhood is often noted as the sole clinical symptom [9, 17–21]. Various associated malformations have also been observed, and these were found in such as 84.5% of cases from a series of 45 CS patients, where 23 patients were *MNX1* mutation carriers [22].

About 80% of CS cases are at least clinically diagnosed before 16 years of age [7, 23, 24]. However, due to the extensive phenotypic variation, a delayed clinical diagnosis of CS, first revealed in the adult, has been frequently reported [25–30]. Even if mutational analysis cannot identify a pathogenic *MNX1* variant, the diagnosis of CS can be established clinically. However, the detection of a pathogenic *MNX1* mutation is helpful, especially in case of an atypical clinical presentation confirmation of the diagnosis by detection of a pathogenic *MNX1* variant would be most helpful.

Many mutation-positive individuals have been regarded as asymptomatic and, as mentioned above, constipation is often noted as the sole finding in relatives of CS patients [31–34]. Other patients showed only chronic intestinal pseudo-obstruction or müllerian duct anomalies, features that can also be associated with the complete CS spectrum [12, 35]. However, evidence of additional sacral anomalies is often noted on X-ray, CT or MRI in these patients [13, 16, 17]. As an imaging procedure for affected patients and their parents, we recommend first spinal and pelvic ultrasound, then sacral x-ray, and in case of questionable or incomplete findings, MRI. Reviewing the clinical features present in 205 CS patients, Lynch et al. observed only two individuals who were mutation carriers (1%) with no signs of the disease on X-ray [13]. As can be seen from the extensive literature review (Additional file 1: Table S2), a total of 25 cases with a proven *MNX1* mutation turned out to be negative for any CS feature following extensive physical examination. However, in two patients, constipation was noted and radiological examinations had not been performed in eight of these individuals. Hence, one must carefully differentiate between asymptomatic and unaffected CS relatives. In any situation, we recommend, that the patient's parents also undergo genetic testing in order to identify mutation carriers among parents. Together with the standardized approach of screening parents for signs of CS, it is possible to obtain true figures of prevalence and penetrance.

The variability of CS has so far prevented an estimation of its true prevalence [15, 31]. However, the OrphaNet entry (ORPHA 1552) for rare diseases lists a prevalence of 1–9/100,000 for CS without referring to the original source.

In several studies, no difference between male and female frequency has been noted [17, 22, 36, 37], whereas other reports listed a higher frequency of CS females [3, 12, 33, 38, 39]. Haga et al. estimated a CS female-to-male ratio in adult cases of 6:1 and of 2:1 in pediatric cases, which may rely, at least in part, on the delayed diagnosis of mildly affected patients [40]. To obtain a more reliable value, we listed the sex of all mutation-positive CS cases and their tested family members from our literature survey (Additional file 1: Table S1). In total, information was given for 308 patients (179 [58.1%] CS females and 129 [41.9%] CS males), resulting in a female-to-male ratio of 1.39:1. This slightly higher incidence rate in females may reflect their gynecological and urological symptoms, possibly representing an ascertainment bias [13]. On the other hand, female predominance would not be expected if CS is exclusively inherited in an autosomal dominant fashion.

### Genotype–phenotype correlation

The various different mutations found within the *MNX1* gene lead to comparable phenotypes. However, there is not only a wide variability among related patients but also between unrelated patients sharing the same mutation [41]. Consequently, no obvious genotype–phenotype correlation has been noted yet. As suggested by various authors [11, 16, 17, 40], this might be explained by the effects of other genes/proteins modifying *MNX1* expression and/or protein function. On the other hand, as among *MNX1* mutation carriers, haploinsufficiency is the most likely cause of the disease [16], the degree of transcriptional activity of the non-mutated *MNX1* allele may vary between CS patients. Also, sequence variants in the long non-coding *MNX1* antisense RNAs, *MNX1-AS1* and *MNX1-AS2* [42, 43], or differences in their expression, may alter the phenotypic outcome.

Recently, Costanzo et al. analyzed the data of 45 CS patients and their findings indicated a correlation with a more severe phenotype when a *MNX1* mutation is present [22]. These authors concluded that *MNX1* seems the main gene responsible for the expression and the severity of the CS triad, while the associated anomalies appear to be mainly determined by other genes.

### Pathogenic concept

Currarino et al. [2] were the first to speculate, that the features of CS rather represent a syndrome complex and share the same embryonic pathway. In human tailbud, the neural tube, notochord, somites and hindgut arise from pluripotent epiblast cells at around Carnegie stage 12 (CS12) corresponding to an assigned embryonic age of 29–31 days [44]. A primary defect in the caudal notochord may prevent some of these cells to migrate from the primitive node. As proposed by several authors, this will lead to a defect at the caudal most aspect of notochord or splitting of notochord and the formation of a fistula between the gut ventrally and the neural elements dorsally or vice versa [2, 15, 16, 45]. Gupta et al. also suggested that failure of some epiblast cells to migrate from primitive node would lead to remnants at the primitive streak, which may persist in the sacrococcygeal region as a teratoma [45].

Experimental evidence for these theories emerged from the detection of *MNX1* in the tailbud of *Xenopus laevis* embryos at tadpole stages [46, 47]. However, human embryonic expression studies were less convincing. Ross and colleagues detected *MNX1* expression in the sacral region at CS19 (embryonic age 45–47 days) but found a pronounced expression in the anterior horn regions along the spinal cord [47]. A more extended analysis was reported by Hagan et al. [16], who investigated embryonic *MNX1* expression from CS12 to CS21, corresponding to embryonic age 29–55 days [44]. Here, the authors were consistently unable to detect *MNX1* expression in tail bud notochord from CS12 onward or in more anterior regions of the notochord. *MNX1* expression was observed in developing human foregut (pharynx, esophagus, stomach), in the basal plate of hindbrain and in developing motor neurons throughout the length of the developing spinal cord. The latter observation supported the findings in *Xenopus laevis* that *MNX1* is a marker for motor neurons [46] and its determined role in cell proliferation and apoptosis in *Drosophila* neuronal cells [48]. *MNX1* has also an essential role in motor neuron differentiation and in the consolidation of motor neuron identity [49], albeit CS patients do not show any motor neuron dysfunction.

Hagan et al. detected *MNX1* expression also in early human pancreatic development, at the time before fusion of the dorsal and ventral buds [16]. Contrary to mouse, *MNX1* expression was here observed throughout CS12-21, with no obvious differences in intensity between the dorsal and ventral buds after CS15. In mouse, transient *Mnx1* expression was seen in dorsal and ventral pancreatic buds from embryonic day (e) 8 to e9-e10 and in mature β-cells of the islets of Langerhans [50, 51]. These observations were verified by Sherwood and colleagues, who revealed, that mouse *Mnx1* is expressed throughout the anterior–posterior axis of the endoderm and here, forms a dorsal–ventral gradient at e9.5 [52]. Li and Edlund have also shown that a tight temporal regulation of *MNX1* expression is necessary, and that a complete loss of expression resulted in a block of the initiation of the dorsal pancreatic program, while temporally extended *MNX1* expression led to a complete impairment of pancreatic development [53]. Mice lacking *MNX1* show a selective agenesis of the dorsal pancreas and abnormal islets of Langerhans, whereas a pancreatic agenesis has yet never been observed in CS patients. It has also been speculated that defects in *MNX1* may be causally related to the 200–250 times higher prevalence of sacral agenesis in diabetic pregnancies [54, 55] or in cases with failure of pancreatic development [56]. However, in no such case, a mutant *MNX1* gene has been identified yet.

The main features in CS patients are frequently associated with urogenital and renal anomalies, e.g. hydronephrosis, horseshoe kidneys, congenital single kidney, vesico-uretral reflux, bicornuate uterus or subseptate vagina [2, 25, 57]. Specific *MNX1* caudal mesoderm requirements have so far been detected only in zebrafish. Here, expression of *Mnx2b*, one of three zebrafish *Mnx* genes, is restricted to the nephric mesoderm during somite stages and its knockdown led to proximal pronephric tubule dilatation and impaired pronephric excretion [58]. A requirement for *Mnx1* in the developing kidney and urogenital system has yet not been observed in mutant mice. However, *Mnx1* expression has been found in the mouse genitourinary system at E10.5 [59] and in mouse lower urinary tract [60]. All these *Mnx1* gene activities observed in non-human systems should be further investigated and might help to better understand the caudal mesoderm specific agenesis in CS patients.

Further studies also revealed that *MNX1* acts in the regulation of progenitor cells into mature hematopoietic cells [61, 62]. The finding of increased *MNX1* levels in patients with acute leukemia and colorectal or hepatocellular cancers suggests that MNX1 protein harbors an additional tumor suppressor function [12, 63–65].

Although one might suspect genetic factors that define specific subtypes of CS, those factors have not been identified yet. Accordingly, there has no phenotype-genotype correlation been established so far.

Taken together, all these observations demonstrate considerable differences in human and murine development. Factual, none of these models reflects the human situation in terms of timing and intensity of *MNX1* expression and the induced mutant phenotype.

### Mouse models for CS

As mentioned above, *Mnx1* knockout mice described so far showed anomalies in pancreatic and motor-neuron development but did not reflect any feature of CS, in particular disruption of sacral or hindgut development. Moreover, *Mnx1*^−/+^ heterozygote mice showed no discernible phenotype [49–51, 66]. In view of these interspecies differences Martin Catala proposed, that either mouse Mnx1 protein has different functions compared with its human ortholog or that human *MNX1* mutations lead to a gain of function instead of being true null mutations [55].

A sole mouse model, resembling CS, was established by Liu and coworkers in 2003 [67]. Here, administration of the highly lipophilic, long-acting synthetic aromatic retinoid etretinate, a potent teratogen affecting the tail bud, on gestation day (E) 9, proved to be capable to induce a CS phenotype. All treated embryos exhibited a presacral mass (which turned out to be an anterior sacral meningocele) and a sacral defect, with anorectal malformations observed in about ¾ of the fetuses. These authors provided evidence, that anorectal malformations, anterior sacral meningocele and sacral defects share the same embryonic pathway. However, the gene(s) targeted by etretinate remain(s) to be elucidated.

#### Pcsk5

Szumska and colleagues identified an ethylnitrosourea-induced recessive p.Cys470Arg mutation in the proprotein convertase subtilisin/kexin type 5 (*Pcsk5*) gene [68]. This mutation induced a pleiotropic effect resulting in cardiac, anorectal, tracheoesophageal and anteroposterior patterning defects, as well as exomphalos, hindlimb hypoplasia, a presacral mass, renal and palatal agenesis, and pulmonary hypoplasia. Since these phenotypic features resembled those seen in VATER/VACTERL association (OMIM% 192350), caudal regression syndrome (OMIM #600145) and CS, the authors coined the term Vcc for this *Pcsk5* mutation. It could also been shown, that Vcc caused ectopic *Mnx1* expression in the ventral aspect of the murine tail bud and a reduced expression of several *Hox* genes [68].

#### Gdf11

As growth/differentiation factor 11 (Gdf11) was shown to be cleaved and activated by Pcsk5, Szumska et al. also investigated *Gdf11*-deficient mouse embryos [68]. At E15.5 all embryos showed an absent tail, palatal and renal agenesis and abnormal anorectal anatomy. Moreover, these embryos had either an intra-abdominal or an extra-abdominal mass arising from the spinal cord and some showed exomphalos. Hence, *Gdf11*-deficiency phenocopies the *Pcsk5* Vcc variant to a large extent. In this context, Tsuda et al. administered teratogenic doses of all-trans retinoic acid at E9 to pregnant mice [69]. This treatment inhibited *Pcsk5* and *Gdf11* expression in the hindgut at E12 and E18 and also resulted in a phenotype resembling caudal regression syndrome and CS. Most of these embryos showed anorectal anomalies and a short tail, as well as sacral malformations, tethered spinal cords and presacral masses.

#### T-box transcription factor brachyury (T)

The idea of the *T*- (*Brachyury*) gene as a candidate for sacral agenesis emerged from the observation of tail anomalies in the *T*-deficient mouse, resembling human spinal defects [70]. As homozygous *T*^*−/−*^ mice die between E9.5 and E10.5 with an overall loss of mesoderm, Pennimpede et al. used in vivo knockdown experiments [71]. Here, at E12.5, the embryos displayed axial skeletal defects and urorectal malformations resembling murine uro-rectal-caudal syndrome and human caudal regression syndrome phenotypes.

### *MNX1*, the major human CS disease gene

The locus for CS was initially mapped to chromosome 7q36 [15] and here, the *MNX1* gene (earlier termed *HLXB9*), encoding motor neuron and pancreas homeobox protein 1 (MNX1, earlier termed HB9), was first described by Harrison et al. [62]. The gene locates to 7q36.3, consists of three exons and, according to UniProtKB entry P50219, the resultant protein comprises 401 amino acids. The use of an alternate 5′-exon may result in a shorter isoform 2 (189 residues), displaying a distinct aminoterminus (see below). MNX1 functions through sequence-specific DNA binding and transcription factor activity, with its homeodomain encoded by amino acids 241–300 (PROSITE annotation). A further significant feature of MNX1 is a polyalanine stretch of variable length (12–18 residues) starting at amino acid 121 (see below).

Around 120 different heterozygous pathogenic *MNX1* single nucleotide variants have yet been described, whereas a large deletion or a complex rearrangement, involving chromosome 7q36, is less commonly involved in the formation of CS. Here, we performed an intensive literature survey for reports describing CS cases and focused on independent patients with a proven genetic anomaly. Also, patients with apparently multiple listing were excluded (Additional file 1: Table S1). This search revealed, that *MNX1* gene mutations or complete gene deletions have been observed in a total of 168 independent CS cases yet. Of these, 108 (64.3%) were found in multiple affected families, whereas 55 (32.7%) represent sporadic cases. For five mutation-positive cases (3.0%), the sporadic or familial occurrence was not mentioned. Altogether, these studies investigated 296 unrelated cases of CS, including 117 familial and 172 sporadic cases; for seven patients no status was given. No mutation was found in nine (7.7%) familial and 117 (68%) sporadic cases. Hence, overall mutation analysis was successful in only 57.4% of all CS patients investigated. Consequently, aside from pathogenic *MNX1* mutations residing in introns, promoter or other transcriptional relevant elements that were not detectable by the methods applied, one might expect further genes to be involved in the etiology of CS.

So far however, other chromosomal or gene anomalies were identified in only five patients (see below). Here, a CS phenotype was either associated with partial trisomy of chromosomes 13q and 20p [72], a duplication 3q26.32-q27.2 [73], a duplication 3q26.31-q29 with deletion 9p24.3-9p23 [74], a functional disomy Xp with deletion Xq13.2-q28 and duplication 3q25.33-q29 [75], or excessive heterochromatin in chromosome 9 (9qh) [76].

### Homozygous *MNX1* mutations

Three different *MNX1* point mutations, present in the homozygous state, have been reported yet. Here, two homozygous germline *MNX1* mutations (p.Phe248Leu and p.Phe272Leu) were identified in two unrelated consanguineous patients with permanent neonatal diabetes mellitus (PNDM; OMIM #606176) [77, 78]. Both patients showed severe intrauterine growth retardation and one female (p.Phe248Leu) died at early age. Whereas p.Phe272Leu was associated with isolated PNDM without any signs of CS, the female harboring the p.Phe248Leu variant also showed sacral agenesis and high imperforate anus, hence cardinal features of CS, as well as severe neurological complications and hypoplastic lungs. The heterozygote parents of the patient with the p.Phe248Leu showed no phenotype. Functional characterization of the p.Phe248Leu variant revealed a normal localization of the mutant protein in the nucleus but a lack of MNX1 phosphorylation [79]. The phenotypic differences caused by these two homozygous mutations might be due to their type and/or location but again reflect the striking variability caused by *MNX1* mutations. As PNDM is not a feature of CS and a normal pancreas is present in heterozygous mice [50], one may speculate, that one functional copy of the *MNX1* gene is at least sufficient for normal human pancreatic development and glucose homeostasis.

A third homozygous amino acid substitution, affecting the initiator codon (p.Met1Thr), was reported in a fetus, also born to consanguineous parents [80]. Whereas the heterozygote parents showed no phenotype, the fetus died within minutes of birth with intrauterine growth retardation, cleft palate, and the CS features anal imperforation, scrotal agenesis and agenesis of the lumbar and sacral spine and spinal cord. Three further newborns of these parents also died with a CS phenotype. The authors concluded, that p.Met1Thr represents a hypomorphic variant, being tolerated in heterozygous carriers but causing severe defects in the homozygous state [80].

Noteworthy, the probability of being loss-of-function intolerant (pLI) score of *MNX1* is 0.79 (https://gnomad.broadinstitute.org/) indicating haploinsufficiency as a potential mechanism. Although the figure does not meet the generally accepted threshold of > 0.9 for intolerant genes, the lower value of *MNX1* might be explained by the low numbers on which the pLI score of *MNX1* is based (indicated by the wide 90% confidence interval of the observed/expected score of 0.04–0.5). In regard of homozygous variants, in gnomAD no truncating variant is reported homozygously.

### MNX1 polyalanine polymorphism

A part of *MNX1* exon 1 encodes a polyalanine stretch, first described to comprise 16 consecutive alanine codons [62]. This coding region however, includes a variable CGC (or GCC) repeat, with e.g. (CGC or GCC)_8_ resulting in a total length of 13 alanine residues, or (CGC or GCC)_12_ finally leading to 17 alanines. Belloni et al. determined that the (CGC)_11_ allele was the most common (90.23%) in the general Caucasian population [3]. The frequencies of other alleles observed by this group was: 0.6% (CGC)_8_, 7.47% (CGC)_9_ and 1.7% (CGC)_12_. Holm et al. found comparable values in a Norwegian CS family and unaffected relatives and reported a further rare allele, (CGC)_7_ [81]. Significant differences in allele distribution were reported in healthy Chinese individuals, where the frequency of (CGC)_11_ was “only” 50% [21]. Here, other alleles showed frequencies of 0.4% (CGC)_8_, 33% (CGC)_9_, and 16.2% (CGC)_12_, respectively. These authors also found a (CGC)_13_ allele, being present on 0.4% of the chromosomes, analyzed from Chinese individuals. Similar frequencies were observed in the Japanese and Yoruba population [21]. Hence, the length of the polyalanine stretch can at least vary from 12 to 18 residues and this length variation has yet not been associated with any phenotype [3, 12, 21, 33]. In that context, the finding of a (CGC)_12_ allele was neither a novel finding nor seems to be responsible for CS in a Taiwanese female reported by Lin et al. [82].

To our knowledge, only one CS patient with a (CGC)_n_ contraction has been reported [12]. In this case, the genotype (CGC)_5_/(CGC)_11_ was observed and the heterozygous (CGC)_5_ allele, never found in controls, was considered responsible for the disease. However, one may also speculate about a critical threshold beyond which a triplet expansion in *MNX1* is pathologic. Disease-causing polyalanine expansion mutations have yet been identified in nine genes (*HOXA13*, *HOXD13*, *SOX3*, *FOXL2*, *PHOXB2*, *RUNX2*, *ARX*, *ZIC2*, and *PABPN1*), all except *PABPN1* encoding transcription factors [83, 84]. As outlined in these reviews, polyalanine expansions cause a loss-of-function and/or mild gain-of function via promotion of misfolding and formation of aggregates of the resultant protein. As such mutant alleles have not been identified in CS patients yet, one may assume a more severe pathological impact of *MNX1* polyalanine expansions on early developmental processes, thereby leading to early embryonic death.

### Expression of an alternative *MNX1* transcript?

As mutation analysis failed to detect *MNX1* mutations in about 43% of all CS patients investigated thus far, the presence of an expressed isoform might explain at least some of these *MNX1* mutation-negative cases. Indeed, UniProtKB lists a second predicted shorter MNX1 isoform (P50219-2), that lacks the N-terminus, present in the 401 amino acids long canonical sequence (P50219-1). This transcript should arise through the use of an alternative exon 1, spliced to the common exons 2 and 3. As this exon 1 encodes a different 19 amino acid long N-terminus, not investigated by the common methods applied, mutations in this stretch may have been missed.

To address this question, we have searched for the presence of the shorter isoform in cDNA samples obtained from blood, saliva and hippocampal tissue. Primers were designed to cover part of the novel exon 1 (5′-CGCTGCCCTCCTCTGGAAGG-3′) and to span the canonical exons 2 and 3 (5′-AATCTTCACCTGGGTCTCGGTGAG-3′). However, these analyses failed to detect mRNA isoform P5019-2 in multiple assays (data not shown), indicating that this mRNA is not substantially expressed at least in these tissues. Hence, it seems to be unlikely, that mutations in this predicted shorter *MNX1* isoform contribute to CS.

### Analysis of further CS candidate genes

In patients with CS, no mutation in the genes encoding *PCSK5* or *GDF11* has been reported and, to our knowledge, no study analyzing these genes in a CS cohort, negative for *MNX1* mutations has yet been performed. However, some other genes, either affected by a deletion of 7q36, or with known phenotypic consequences in mouse models have been investigated.

#### SHH

Sonic hedgehog (SHH) is an endoderm-derived signaling molecule. Although mutant mice with various defects in SHH signaling only displayed a spectrum of anorectal malformations [85], it might also be involved in caudal regression. As the *SHH* gene locates to chromosome 7q36.3, the critical region initially linked to CS [15], Seri et al. investigated *SHH* in a large CS multiplex family with eight affected members, seven further sporadic CS cases and an additional 15 patients with anorectal malformations and sacral hypodevelopment [86]. No mutation was observed, making an involvement of *SHH* in these diseases rather unlikely. This assumption has been corroborated by Coutton et al. [87]. These authors reported a patient with CS and a microform of holoprosencephaly, who carried a 2.7 Mb deletion in 7q36.3 without affecting the *SHH* gene.

#### T (Brachyury)

A study of 28 patients with sacral agenesis with anorectal atresia revealed, that the coding region of the *T*-gene is highly polymorphic [88]. However, Papapetrou et al. detected one rare heterozygous p.Ala338Val variant in a female patient that was transmitted by an apparently unaffected mother [88]. This variant was also detected in three further heterozygous patients, presenting with either sacral agenesis, Klippel-Feil syndrome or multiple cervical and thoracic vertebral malformations [89]. Again, the variant had been inherited from a clinically unaffected parent in all three cases. The p.Ala338Val substitution has been deposited with no. rs117097130 in dbSNP (Build 146) and the EXAC_0.3 database lists its occurrence with a minor allele frequency of 0.004835 (A) in a sample of 121.412 individuals. Hence, based on current knowledge, its contribution to the associated phenotypes may not be excluded.

This assumption is supported by another study presenting evidence, that homozygous *T*-mutations cause a syndrome consisting of sacral agenesis, abnormal ossification of the vertebral bodies and a persistent notochordal canal [90]. The authors identified a homozygous p.His171Arg mutation in three consanguineous families of the same ethnic background. The mutant protein showed reduced DNA binding activity and a decrease in activation activity of T binding sites. As outlined by Postma et al., this observation suggests, that screening for the ossification of the vertebrae is warranted in patients with sacral agenesis to evaluate the possible causal involvement of the *T* gene [90]. Further support arises from the finding of a multiplex consanguineous Saudi family with four patients born with isolated myelomeningocele [91]. The here identified novel p.Gly156Cys *T* variant was found in the homozygous state in all four patients but also in three apparently non-penetrant siblings, although MRI/CT examination performed in one of them revealed the presence of spina bifida oculta. These observations allow for potentially reduced penetrance and support the assumption of an involvement of the *T* gene in the etiology of CS.

#### Cytogenetic findings and copy number variations

Chromosomal anomalies, not involving region 7q36, have rarely been associated with CS or a CS-like phenotype. In one patient with partial sacral agenesis, Anderson-Shotwell and Wilson found a de novo deletion of chromosome 18p [92]. Nagai et al. reported a CS patient with partial trisomy of chromosomes 13q and 20p [72]. Analyzing six patients with pure interstitial or terminal 6q deletions, Titomanlio et al. mapped a locus for sacral/anorectal malformations to a 0.3 Mb critical region at 6q25.3 [93]. With the four genes *SYNJ2*, *SERAC1*, *GTF2H5* and *TULP4*, this region harbors no obvious candidate for CS. However, the *MNX1* gene had not been analyzed in all these patients. In a *MNX1* mutation negative patient, Bevanda et al. detected an excess of heterochromatin in chromosome 9 (9qh) in the patient and his asymptomatic father [76]. Heterochromatin constitutes highly compacted chromatin with regions of silenced DNA and contains hypoacetylated histones and high levels of DNA methylation. Hence, a more severe imbalance in these DNA modifications might have contributed to CS in the patient compared to his father.

In a more recent report, Dworschak et al. detected a pure 7.9 Mb de novo duplication 3q26.32-q27.2 in an *MNX1* mutation-negative CS patient [73]. This male patient not only showed the classical CS triad with anal atresia, presacral teratoma and hemisacrum but presented additional features (developmental delay, CNS malformations, facial dysmorphism), frequently detected in duplication 3q syndrome. Aljeaid and coworkers lately described a CS female patient with additional congenital heart defects, generalized hypotonia, global developmental delay, dysmorphic facial features and dysplastic ears [74]. These authors also detected a de novo terminal duplication 3q. Here, the duplicated region turned out to be larger, comprising 24.6 Mb from 3q26.31-q29, and the patient also carried a de novo 12.5 Mb terminal deletion from 9p24.3-9p23. Moreover, Peterson et al. described a female with a combination of functional disomy Xp, deletion Xq13.2-q28 and duplication 3q25.33-q29 [75]. This patient presented with features of CS (sacral teratoma and neurogenic bladder) as well as primary ovarian insufficiency, sensorineural hearing loss and intellectual disability.

All these latter observations suggest, that a dosis effect of (a) gene(s) in dup(3q) may explain the phenotype in some patients negative for *MNX1* mutations or 7q36 deletions involving the *MNX1* locus.

#### Whole exome sequencing data

Whole exome sequencing has been performed in three patients with sporadic CS and their parents [94]. Here, Holm et al. also analyzed a custom-made region on chromosome 7 to detect variants in regulatory elements in non-coding regions around the *MNX1* gene and additionally investigated biopsy material from the removed presacral mass in one case [94]. No sequence variant was shared between the three patients in non-coding regions surrounding *MNX1* or in other transcription factor genes, known to interact with *MNX1*. However, the authors prioritized three variants that might be of relevance to CS [94]. One patient carried a de novo p.Val126Ile variant in *ETV3L*, encoding the transcription factor ETS variant 3 like, implicated in tissue development and the equilibrium between proliferation and differentiation [95]. The second patient also carried one de novo variant, affecting the acceptor splice site of exon 11 of the *NCAPD3* gene, coding for condensin-2 complex subunit D3. This protein is a regulatory subunit of the condensin-2 complex, which establishes mitotic chromosome architecture [96]. In the third CS patient, a p.Arg55Leu variant was identified in *ARID5A*. Here, the encoded protein, AT-rich interactive domain-containing protein 5A, is a sequence-specific transcription factor with recognized promoter targeting functions and important roles in development and differentiation [97]. This variant however, was transmitted from the unaffected mother.

Most recently, Han et al. found four likely pathogenic de novo variants in three sporadic CS patients [39]. A *CDH2* missense variant (p.Arg151Ser) was detected in one of these patients. This gene encodes the calcium-dependent cell adhesion protein cadherin-2, a cell surface transmembrane protein with a vital structural and functional role in the intercalated disc, involved in cell-to-cell adhesion. So far, mutations in this gene have been associated with arrhythmogenic right ventricular cardiomyopathy [98]. Another patient carried a 4 bp deletion (p.Ile541Ile*fs*12) leading to a frameshift variant of the *ITIH2* gene. *ITIH2* codes for the inter-alpha-trypsin inhibitor heavy chain H2, a protein from a family of structurally related plasma serine protease inhibitors playing a key role in extra-cellular matrix biology and tumor progression [99]. In a third patient, two novel *HOXB4* variants (p.Lys16Asn) and *TLE4* (p.Ser650Leu) gene were found. Homeobox protein Hox-B4 is a sequence-specific transcription factor with diverse roles in embryonic development and the regulation of adult stem cells. As outlined in Morgan et al., it intriguingly can act in opposite ways when expressed by different cells, promoting the proliferation of stem cells whilst activating the apoptotic pathway in some embryonic structures [100]. The transducin-like enhancer protein 4, encoded by *TLE4*, binds to various transcription factors and acts as a transcriptional corepressor in regulating e.g. Wnt, Notch and transforming growth factor-ß signaling [101].

At least some of these genes represent apparently interesting candidates for an involvement in CS etiology, however, their involvement remains to be elucidated.

#### Regulatory sequences

Conserved non-coding sequences (CNEs) are known to act as expression regulators and may function either as an enhancer or repressor of transcription, depending on the regulatory protein(s) bound to it [102]. In that context, Woolfe and colleagues have tested various CNEs for enhancer activity in vertebrate development [103]. Three of these CNEs, located distal or proximal to *MNX1*, indeed directed gene expression to skin/enveloping layer, skeletal muscle or spinal cord in zebrafish embryos. These findings suggest that a sequence variation in one of these CNEs may contribute to CS in a so far unknown way.

## Conclusion

The yet suggested testing strategy for CS only includes *MNX1* sequence- and MLPA-analysis [104]. In contrast we suggest whole-exome sequencing and molecular karyotyping (CNV analysis) in order to identify possible further candidate genes involved in the formation of CS. As CS exhibits an extreme variable expressivity with many asymptomatic cases, it is often overlooked or misdiagnosed, a fact that yet opposes the chance to more precisely define its true incidence.

## Supplementary Information

**Additional file 1.** **Supplemental Table 1.** CS patients with reported heterozygous MNX1 mutations or associated gene/chromosomal anomalies. **Supplemental Table 2.** Cases with heterozygous MNX1 or other mutations negative for CS features.

## Data Availability

Not applicable.

## Abbreviations

CNEs: Conserved non-coding sequences
CS: Currarino syndrome
