# Supplementary material for: Currarino syndrome: a comprehensive genetic review of a rare congenital disorder
Source: Orphanet J Rare Dis. 2021 Apr 9;16:167. doi: 10.1186/s13023-021-01799-0 (PMC8034116; doi:10.1186/s13023-021-01799-0)
Supplement: Supplementary file 1 — Additional file 1. Supplemental Table 1. CS patients with reported heterozygous MNX1 mutations or associated gene/chromosomal anomalies. Supplemental Table 2. Cases with heterozygous MNX1 or other mutations negative for CS features. [file 13023_2021_1799_MOESM1_ESM.docx]

**Additional file 1: Table S1.** CS patients with reported heterozygous *MNX1* mutations or associated gene/chromosomal anomalies

| No. of independent CS cases reported | No. of CS mutation-positive males (m)/ females (f) | | No. of familial (F) and sporadic (S) cases | No. of patients with *MNX1* mutation or gene deletion | No. of patients with other chromosomal or gene anomalies | Reference no. (literature for multiple mentioning of patients) | |
| --- | --- | --- | --- | --- | --- | --- | --- |
| 1 with complete CS | f=1 | | S=1 | - | del 7q35-qter | 105 | |
| 10 (one with additional HPE) | m=1/f=6 | | F=4, S=6 | F=3, S=4; 7 (70%) | - | 3 | |
| 28 with complete CS | no data | | F=21, S=7 | F=20, S=2; 22 (79%) | - | 16 (13, 47) | |
| 9 (at least 1 with incomplete CS | m=11/f=12 | | F=4, S=5 | F=4, S=1; 5 (55%) | - | 17 | |
| 1 with incomplete CS | f=1 | | S=1 | - | partial monosomy 7q / trisomy 2p (46,XX/46,XX,add(7q36).ish der(7)(q36),t(2;7)(p22;q36) | 106 | |
| 1 with incomplete CS | m=1 | | S=1 | - | del 7q36.1-qter | 107 | |
| 6 (4 complete CS) | m=3/f=8 | | F=3, S=3 | F=2, S=1; 3 (50%) | - | 20 | |
| 5 with complete CS | 11=m/12=f | | F=5 | 5 nd (100%) | - | 36 | |
| 1 with complete CS | m=7/f=2 | | F=1 | 1 (100%) | - | 108 | |
| 1 with complete CS | no data | | F=1 | 1 (100%) | - | 109 (one familiar case from 47 and 81) | |
| 11 with complete CS | m=5/f=7 | | F=5, S=6 | F=4, S=2; 6 (55%) | - | 21 | |
| 1 with complete CS | m=1/f=5 | | F=1 | 1 (100%) | - | 32 | |
| 1 with complete CS and ventriculomegaly | m=1/f=2 | | F=1 | 1 (100%) | - | 26 | |
| 1 with complete CS | f=1 | | F=1 | 1 (100%) | - | 110 | |
| 4 with complete CS | m=3/f=8 | | F=2, S=2 | F=2 (50%) | - | 111 | |
| 1 with incomplete CS | f=1 | | S=1 | 1 (100%) | - | 112 | |
| 1 with complete CS | m=1 | | F=1 | 1 (100%) | - | 113 | |
| 50 with at least two CS features | m=21/f=31 | | F=20, S=30 | F=19, S=4; 23 (46%) | S=1 [46,XY,inv7(p15.1;q34)]  S=1 [46,XY,der(7)t(7;12)(q36.12;q23.21).pat]  S=1 [46,XX,ins(4;7)(q25;q31.3q21.3)] | 12, 14 | |
| 4 with complete CS | m=5/f=2 | | F=1, S=3 | F=1, S=3; 4 (100%) | - | 41 | |
| 1 with complete CS and treatment- resistant meningitis | f=1 | | F=1 | 1 (100%) | - | 114 | |
| 1 with incomplete CS | f=1 | | ? | 1 (100%) | - | 57 | |
| 1 with complete CS and microcephaly, growth-impairment, sensorineural deafness and facial dysmorphism | f=1 | | S=1 | - | del 8.8-Mb of 7q36 and dup 10.3-Mb of 7q34-q35 | 115 | |
| 1 incomplete CS | f=1 | | F=1 | 1 (100%) | - | 28 | |
| 4 (all complete CS) | m=5/f=1 | | F=1, S=3 | F=1, S=1; 2 (50%) | - | 116 | |
| 2 with complete CS | m=5/f=3 | | F=2 | 2 (100%) | - | 117 | |
| 1 with complete CS | m=1 | | F=1 | 1 nd (100%) | - | 118 | |
| 1 with complete CS | m=1/f=1 | | F=1 | 1 (100%) | - | 119 | |
| 9 with complete CS | m=14/f=15 | | 3=F, S=6 | F=3 (100%) | - | 94 (two familiar cases from 47) | |
| 1 with complete CS | m=1/f=2 | | F=1 | 1 nd (100%) | - | 120 | |
| 28 (9 complete CS) | m=8/f=21 | | F=8, S=18, nd=2 | F=6, S=6, nd=2; 14 (50%) | - | 33 | |
| 8 with complete CS (2 with additional cerebral anomalies) | no data | | S=8 | 1 (12.5%) | del 7q36 | 37 (several cases had been reported in 81) | |
| 1 with complete CS and HPE microform | m=1 | | S=1 | - | del 2.7-Mb of 7q36.3 (excluding *SHH*) | 87 | |
| 4 with complete CS | m=4/f=2 | | F=1, S=3 | F=1 (100%) | S=1 (del 20-Mb of 7q32-qter and dup 0.96-Mb of 13q34-qter)  S=1 (del 9.2-Mb of 7q36.1-qter and dup 3.5-Mb of 14q32.32-qter)  S=1 (del 4.4-Mb of 7q36.2-qter and dup 17.2-Mb 7q33-q36.2) | 104 | |
| 2 (1 complete CS) | f=2 | | F=1 | F=1 (100%) | - | 121 | |
| 4 with complete CS | m=3/f=2 | | F=2, S=2 | S=2 (50%) | - | 122 | |
| 45 (20 with complete CS) | m=1/f=1 further data not given | | F=13, S=30, 2 nd | 38 tested  F=13, S=13; 26 (68%) | S=2 (7q microdeletion including *MNX1* and *SHH*)  S=2 larger 7q deletion | 22 | |
| 2 with complete CS | m=2 | | F=1 | F=1 (100%) | - | 123 | |
| 25 (21 complete CS) | m=2/f=7 | | F=1, S=24 | F=1, S=6; 7 (28%) | S=1 (del 5.1-Mb of 7q36) | 38 | |
| 1 with incomplete CS and microcephaly | f=1 | | S=1 | - | del 4.15 Mb of 7q36.2-q36.3 including *SHH* | 124 | |
| 1 with complete CS | f=1 | | ? | 1 (100%) | - | 125 | |
| 2 with incomplete CS | m=2/f=1 further data not given | | F=2 | F=2 (100%) | - | 11 | |
| 1 with incomplete CS | m=1 | | ? | 1 (100%) | mosaicism with r(7), monosomy 7 and duplicated r(7) | 126 | |
| 16 (14 with complete CS) | m=4/f=14* | | F=7/S=9* | F=7 (100%)/ S=2(22%)* | - | 39 | |
| **No involvement of *MNX1*** | | | | | | |  |
| 1 with complete CS | m=1 | S=1 | | - | partial trisomy 13q and partial trisomy 20p | 72 | |
| 1 with complete CS and developmental delay, CNS malformations and facial dysmorphism | m=1 | S=1 | | - | dup 3q26.32-q27.2 | 731 | |
| 1 with complete CS and congenital heart defects, generalized hypotonia, global developmental delay, dysmorphic facial features and dysplastic ears | f=1 | S=1 | | - | dup 24.6 Mb of 3q26.31-q29 and del 12.5 Mb of 9p24.3-9p23 | 74 | |
| 1 with incomplete CS and primary ovarian insufficiency, sensorineural hearing loss and intellectual disability | f=1 | S=1 | | - | dup 3q25.33-q29 with functional disomy Xp and del Xq13.2-q28 | 75 | |
| 1 with incomplete CS | m=1 | S=1 | |  | high heterochromatin in chromosome 9, 46 XY, 9qh | 76 | |

nd, no details given; *data have been changed according to the published data, where several patients, suggested to be sporadic, turned out to be familiar.

Additional references are as follows:

105. Masuno M, Imaizumi K, Aida N, Tanaka Y, Sekido KI, Ohhama Y, Nishi T, Kuroki Y. Currarino triad with a terminal deletion 7q35->qter. J Med Genet. 1996;33:877-8.

106. Le Caignec C, Winer N, Boceno M, et al. Prenatal diagnosis of sacrococcygeal teratoma with constitutional partial monosomy 7q/trisomy 2p. Prenat Diagn. 2003;23:981-4.

107. Horn D, Tönnies H, Neitzel H, Wahl D, Hinkel GK, von Moers A, Bartsch O. Minimal clinical expression of the holoprosencephaly spectrum and of Currarino syndrome due to different cytogenetic rearrangements deleting the *Sonic Hedgehog* gene and the *HLXB9* gene at 7q36.3. Am J Med Genet Part A. 2004;128A:85-92.

108. Urioste M, del Carmen Garcia-Andrade M, Valle L, et al. Malignant degeneration of presacral teratoma in the Currarino anomaly. Am J Med Genet Part A. 2004;128A:299-304.

109. Verlinsky Y, Rechitsky S, Kuliev A, Schoolcraft W. Preimplantation diagnosis for homeobox gene *HLXB9* mutation causing Currarino syndrome. Am J Med Genet Part A. 2005;134A:103-4.

110. Fleury J, Picherot G, Crétolle C, Podevin G, David A, Caillon J, Roze JC, Gras-le Guen C. Currarino syndrome as an etiology of a neonatal *Escherichia coli* meningitis. J Perinatol. 2007;27:589-91.

111. Kim IS, Oh SY, Choi SJ, Kim JH, Park KH, Park HK, Kim JW, Ki CS. Clinical and genetic analysis of HLXB9 gene in Korean patients with Currarino syndrome. J Hum Genet. 2007;52:698-701.

112. Liang Y, Wang J, Cai W. Clinical features and *HLXB9* gene mutation of a sporadic Chinese Currarino’s syndrome case. J Pediatr Surg. 2007;42:E27-30.

113. Volk A, Karbasiyan M, Semmler A, Todt U, Urbach H, Klockgether T, Linnebank M. Adult index patient with Currarino syndrome due to a novel *HLXB9* mutation, c.336dupG (p.P113fsX224), presenting with Hirschsprung’s disease, cephalgia, and lumbodynia. Birth Defects Res A Clin Mol Teratol. 2007;79:249-51.

114. Kiefer AS, Gupta P, Kirmani S, Schwartz K, Henry N, Fischer PR. Treatment-resistant meningitis leading to the diagnosis of Currarino syndrome. Pediatr Infect Dis J. 2009;28:547-9.

115. Pavone P, Ruggieri M, Lombardo I, et al. Microcephaly, sensorineural deadfness and Currarino triad with duplication-deletion of distal 7q. Eur J Pediatr. 2010;169:475-81.

116. Zu S, Winberg J, Arnberg F, Palmer G, Svensson PJ, Wester T, Nordenskjöld A. Mutation analysis of the motor neuron and pancreas homeobox 1 (*MNX1*, former *HLXB9*) gene in Swedish patients with Currarino syndrome. J Pediatr Surg. 2011;46:1390-5.

117. Markljung E, Adamovic T, Cao J, Naji H, Kaiser S, Wester T, Nordenskjöld A. Novel mutations in the *MNX1* gene in two families with Currarino syndrome and variable phenotype. Gene 2012;507:50-3.

118. Sekaran P, Brindley N. A case of Currarino’s syndrome presenting as neonatal bowel obstruction. J Pediatr Surg. 2012;47:1600-3.

119. Wang Y, Wu Y. A novel HLXB9 mutation in a Chinese family with Currarino syndrome. Eur J Pediatr Surg. 2012;22:243-5.

120. Kim AY, Yoo SY, Kim JH, Eo H, Jeon TY. Currarino syndrome: variable imaging features in three siblings with *HLXB9* mutation. Clin Imaging 2013;37:398-402.

121. Scimone C, Donato L, Rinaldi C, Sidoti A, D’Angelo R. First case of Currarino syndrome and trimethylaminuria: two rare diseases for a complex clinical presentation. J Dig Dis. 2016;17:628-32.

122. Caro-Domínguez P, Bass J, Hurteau-Miller J. Currarino syndrome in a fetus, infant, child, and adolescent: Spectrum of clinical presentations and imaging findings. Can Assoc Radiol J. 2017;68:90-5.

123. Cananzi M, Colavito D, Giorgi B. A case of constipation that passed from father to son. Gastroenterology 2018;155:1317-8.

124. Cococcioni L, Paccagnini S, Pozzi E, et al. Currarino syndrome and microcephaly due to a rare 7q36.2 microdeletion: a case report. Ital J Pediatr. 2018;44:59.

125. Kenevan MR, Smith HM, Olsen DA, Sharpe EE. Ultrasound-assisted combined spinal-epidural anesthesia for Cesarean delivery in a parturient with Currarino triad: A case report. A & A Pract. 2019;12:393-5.

126. Salas-Labadia C, Gomez-Carmona S, Cruz-Alcivar R, et al. Genetic and clinical characterization of 73 pigmentary mosaicism patients: revealing the genetic basis of clinical manifestations. Orphanet J Rare Dis. 2019;14:

**Additional file 1: Table S2.** Cases with heterozygous *MNX1* or other mutations negative for CS features

| mutation carrier without phenotype | unremarkable  on X-ray, CT or MRT | type of *MNX1* mutation | Reference |
| --- | --- | --- | --- |
| male III-7 (family I) | yes | p.Leu240Leu*fs**47 | 47* |
| male I.I and daughter II.6 | yes | splice CAGG to CGGG intron 2 |  |
| mother of patient 060 | not done | p.Thr248Ser | 3 |
| great-aunt of the index patient | not done | p.Leu240Leu*fs**47 | 109 |
| father II.3 (only constipation was noted) | yes | p.Glu283* | 32 |
| mother (family 1) | not done | p.Arg295Trp | 111 |
| uncle (II.7), aunts (II:2, II:4) | not done | p.His260_Gln261delinsLELLELE |  |
| father (family 5) | yes | p.Trp215* | 12 (also reported in 110) |
| maternal aunt of the index patient (family 4) | not done | p.Gln211* | 12 |
| father (family 17) | not done | p.Leu240Leu*fs**47 |  |
| great-grandmother of the index case (family 1) | not done | p.Phe289Ser | 117 |
| father and two nices of the index case (family A) | yes | p.Arg19Thr*fs**37 | 81 |
| three members of family B | yes | del *MNX1* | 37 |
| mother (family 03) (only constipation was noted) | yes | p.Pro27Leu | 33 |
| mother (family 05) | yes | p.Gly103Arg |  |
| father (family 13) | yes | p.Arg292Gly |  |
| mother (family 1)  mother (family 2)  father (family 8) | yes | p.Gly42*  p.Leu223Leu*fs**61  p.Trp288Leu | 39 |

*The carriers reported by Ross et al. [47] should represent those mentioned in Lynch et al. [13] and were also reported in Hagan et al. [16].

References are numbered according to the main text and Additional file 1: Table S1.
